# The association between bariatric surgery and incident rheumatoid arthritis

**DOI:** 10.1093/rap/rkag025

**Published:** 2026-02-18

**Authors:** Helana Jeries, Revital Perlov Gavze, Rula Daood, Fadi Hassan, Liat Lev Shalem, Ahmad Assalia, Mohammad E Naffaa

**Affiliations:** Rheumatology Unit, Galilee Medical Center, Nahariya, Israel; Azrieli Faculty of Medicine, Bar-Ilan University, Safed, Israel; Medical Division, Maccabi Healthcare Services, Tel Aviv, Israel; Medical Division, Maccabi Healthcare Services, Tel Aviv, Israel; Rheumatology Unit, Galilee Medical Center, Nahariya, Israel; Azrieli Faculty of Medicine, Bar-Ilan University, Safed, Israel; Rheumatology Unit, Galilee Medical Center, Nahariya, Israel; Azrieli Faculty of Medicine, Bar-Ilan University, Safed, Israel; Medical Division, Maccabi Healthcare Services, Tel Aviv, Israel; Department of General Surgery, Rambam Healthcare Campus, Haifa, Israel; Rappaport Faculty of Medicine, Technion, Haifa, Israel; Rheumatology Unit, Galilee Medical Center, Nahariya, Israel; Azrieli Faculty of Medicine, Bar-Ilan University, Safed, Israel

**Keywords:** rheumatoid arthritis, obesity, bariatric surgery, rheumatoid arthritis incidence

## Abstract

**Objectives:**

Obesity is considered a controversial risk factor for developing RA and the association between weight change and RA incidence is inconclusive. The aim of our study was to investigate the effect of weight loss after bariatric surgery on the incidence of RA.

**Methods:**

This retrospective cohort study included all patients who underwent bariatric surgery between 1 January 2010 and 31 December 2015 as documented in the Maccabi Healthcare Services (MHS) database. These patients were matched with a control group based on age and sex. The two groups were followed until the earliest of the following: incident RA, leaving MHS, death or end of follow-up by 31 December 2020. Incident RA was identified based on the relevant International Classification of Diseases, Ninth Revision code (714). To further increase the specificity of the diagnosis, RA patients had to additionally fulfil one of the following: RA diagnosis was made by a rheumatologist, being positive for RF or anti-CCP or purchasing at least one DMARD after an RA diagnosis.

**Results:**

Each group included 9583 patients. The mean follow-up period was 7.31 years. Among the 19 166 subjects, 18 incident RA cases were diagnosed, 9 in each group. The mean age of the patients who developed RA was 49.89 years and half of the patients were males. Male gender was associated with an increased risk for developing RA among patients with a BMI >40.

**Conclusion:**

Bariatric surgery was not associated with a reduced risk of incident RA during the 10-year follow-up period.

Key messages

- Obesity is considered a controversial risk factor for RA; however, it is unclear whether weight loss has a protective effect.
- This study shows that weight loss secondary to bariatric surgery is not associated with a reduced risk of developing RA.
- Further studies are needed to better understand the impact of obesity and weight loss on RAs.

## Introduction

RA is a chronic autoimmune inflammatory joint disease that causes chronic damage to joints, resulting in joint deformities, high morbidity and the loss of many working days [1]. Several risk factors, such as female sex, genetic predisposition and smoking, have been shown to increase the risk of developing RA [2]. Obesity has been reported to be a risk factor for developing RA, especially among women, and is associated with increased disease activity and poor treatment response [3–12].

Obesity has been widely investigated as a risk factor for RA [13, 14]. Several case–control and prospective cohort studies have shown an increased risk of RA in obese patients [15, 16], particularly among RF- and anti-CCP-negative female patients [3, 17]. However, other studies have suggested that obesity is not a predisposing factor for RA [18–20].

Regarding RA disease activity, obese patients have more active and severe disease, as indicated by higher inflammatory markers levels, disease activity scores, tender and swollen joint counts, visual analogue scale (VAS) scores and general health scores [7, 21]. It is debated whether obesity is a poor predictor of remission in patients receiving biologics, as studies have shown lower disease activity and remission rates among obese patients who mainly received anti-TNF-α inhibitor therapies but not among those who received other biologics [22–27]. Nevertheless, recently, a report from the Nordic Rheumatic Diseases Strategy Trials and Registries (NORD-STAR) showed that among patients with untreated early RA followed for up to 48 weeks, obesity was associated with a lower likelihood of good treatment response, irrespective of the type of randomized treatment received [28].

Therefore, the question of whether weight loss decreases the incidence of RA is pivotal. Marchand *et al.* [29] examined the association between long-term weight change and RA risk in a large prospective cohort study. Long-term weight gain was strongly associated with increased RA risk in women. Surprisingly, weight loss of >2 kg was associated with an increased RA risk and seropositive RA risk, but this association was not demonstrated in lagged analyses [29].

It has been reported that among RA patients, bariatric surgery and subsequent weight loss are associated with better disease control, as reflected by lower disease activity, decreased serum inflammatory markers and less RA-related medication use [12, 30, 31]. Although the effect of bariatric surgery on RA disease control has been widely reported, the association between bariatric surgery and the incidence of RA has been less studied.

Maglio *et al*. [32] studied the association between bariatric surgery and incident RA among the Swedish population and found that bariatric surgery and its related weight loss were not associated with a decreased risk for developing RA. In addition, changes in BMI at 2 years of follow-up were not associated with the development of RA. The work by Maglio *et al.* [32] has several limitations. First, all patients who were candidates for bariatric surgery were recruited from surgical departments and 14% of the patients in the control group eventually underwent bariatric surgery. Second, the primary endpoint of the Swedish Obesity Subject Study was mortality and not RA diagnosis. A third limitation is that the diagnosis of RA was extracted from the Swedish National Patient Register, making the accuracy of the diagnosis a questionable issue.

Given the association between bariatric surgery and better RA disease control, along with the limitations of the study by Maglio *et al.* [32], we aimed to study the effect of bariatric surgery (and its related weight loss) on RA incidence in a larger cohort of subjects undergoing bariatric surgery.

## Study design and methods

### Data source

The present study analysed data extracted from the Maccabi Healthcare Services (MHS) database. The MHS is a nationwide health plan (payer-provider) representing almost 25% of the population in Israel (2.5 million people). The MHS database contains longitudinal data for a stable population of people since 1993 (with <1%/year moving out). The coverage for healthcare services in Israel is provided by four competing nationwide health maintenance organizations (HMOs), which include both public and private providers of services. The data were automatically collected and included comprehensive clinical and demographic information obtained from patients’ medical records, laboratory data from a single central laboratory and full pharmacy prescription and purchase data for each patient. The MHS uses the International Classification of Diseases, Ninth Revision, Clinical Modification (ICD-9-CM) system, as well as self-developed coding systems, to provide more granular diagnostic information beyond the ICD codes. Medications are coded according to the Israeli coding system with translations to the Anatomical Therapeutic Chemical (ATC) coding system wherever available. Procedures are coded using Current Procedural Terminology (CPT) codes.

### Study population

Patients were included in the bariatric group if they were ≥18 years of age and had undergone their first bariatric surgery after 2010. The subjects in the matched control group were MHS members ≥18 years of age who had a BMI ≥30 and were matched to the bariatric cohorts by age and sex at the index date at a 1:1 ratio.

The index date for the bariatric group was defined as the date of their first bariatric surgery between 1 January 2010 and 31 December 2015 and for the matched group it was defined as the index date of their respective exposed patients in the bariatric surgery group. The follow-up period was defined as the period from the index date until the earliest of the following: incident RA diagnosis, leaving the MHS, death or the end of follow-up by 31 December 2020.

The exclusion criteria were identical for both groups and included previous bariatric surgery or any gastric surgery prior to entry into the cohort and previous purchases of conventional synthetic DMARDs (csDMARDs), biologics or Janus kinase (JAK) inhibitors prior to entry into the cohort and through the first year after the index date. Subjects with a previous positive blood test for either RF >15 U/ml or anti-CCP ≥3 U/ml prior to the index date and 1 year later and patients with <12 months of continuous follow-up in the MHS prior to the index date were excluded from the study. Patients in the control group who underwent bariatric surgery at any point during the follow-up period were not included in the study. We also excluded subjects who developed RA during the first year after the index date, to overcome the risk of surveillance bias.

Research ethical approval was obtained from the Maccabi Healthcare Services Ethics Committee, in accordance with the Declaration of Helsinki (approval no. 20-137-MHS). The requirement for informed consent was waived by the institutional review board of Maccabi Healthcare Services due to the retrospective nature of the study.

### Study outcome and data collection

The primary endpoint of the study was incident RA after the index date and through the follow-up period. The baseline characteristics collected for the study participants included age at index date (years), sex, residence area (north, centre, south, Sharon, Jerusalem and Shfela), smoking status (ever, never, unknown) and socio-economic status (SES) (high, medium, low). SES was defined based on a score ranging from 1 (lowest) to 10 derived for commercial purposes by Points Location Intelligence using geographic information systems (GISs) and data such as expenditures related to retail chains, credit cards and housing. This score is strongly correlated with the SES measured by the Central Bureau of Statistics. A score of 1–10 was categorized by tertiles as low, medium or high.

### Clinical and biochemical assessments

The patients’ dispensed medications included csDMARDs (methotrexate, leflunomide, hydroxychloroquine and sulfasalazine), biologic DMARDs, JAK inhibitors, antidiabetic drugs and statins.

The results of laboratory tests, including haemoglobin A1c (HbA1c), high-density lipoprotein (HDL) cholesterol, low-density lipoprotein (LDL) cholesterol, CRP, ESR, vitamin D and platelet levels, were recorded up to 2 years prior to the index date until the end of follow-up. Chronic comorbidity assessments included diabetes, coronary vascular disease, chronic heart failure, hypertension, chronic kidney disease (CKD), liver cirrhosis and cancer. Obesity was defined as a BMI ≥30, with the closest measurement to the index date occurring up to 5 years prior to the index date.

### RA diagnosis

Incident RA was identified based on the relevant ICD-9 code (714). To further increase the specificity of the diagnosis, RA patients had to additionally fulfil one of the following: RA diagnosis was made and documented by a certified rheumatologist, positive for RF or anti-CCP or purchased at least one DMARD after RA diagnosis.

### Types of bariatric surgery

Sleeve gastrectomy was the predominant bariatric surgery in Israel until 2016 and accounted for 80% of all bariatric surgeries. Gastric bypass was introduced in Israel in 2016 and its use has become more common in recent years.

### Statistical methods

The results were summarized using descriptive statistics [i.e. mean, s.d., median, minimum, maximum and interquartile range (IQR) for continuous variables and number and percentage for categorical variables].

For the univariate comparison between exposed and unexposed patients, the standardized mean difference (SMD) is presented. A standard difference <0.1 indicates a negligible difference in the means or prevalence of a covariate.

Differences between groups were analysed by *t*-tests and chi-squared tests, as appropriate. Kaplan–Meier method estimates of cumulative incidence rates were used to assess the time to incident RA after inclusion. Comparisons between groups were performed by the logrank test. Hazard ratios (HRs) and corresponding 95% CIs for the risk of RA were calculated by Cox proportional hazard models. Two-sided *P*-values <0.05 were considered statistically significant. Statistical analysis was performed using SPSS version 29 (IBM, Armonk, NY, USA).

## Results

### Baseline characteristics of the study population

**Figure 1:**
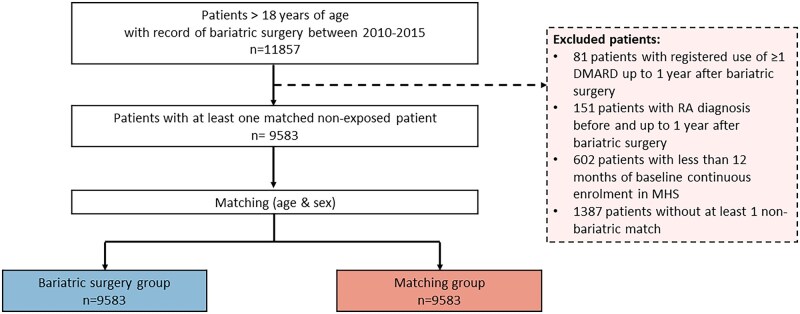
Flow chart of the study population selection

We identified 11 857 patients at the MHS with a history of bariatric surgery between 2010 and 2015. We excluded all patients who were prescribed DMARDs (*n* = 81) or who were diagnosed with RA (*n* = 115) before and up to 1 year after the index date. We also excluded patients with <12 months of continuous follow-up in the MHS (*n* = 602) and patients without an adequately matched control (*n* = 1387). The final cohort included 9583 subjects in each group (Fig. 1).

Table 1 shows the baseline characteristics of the study population. The mean age of the patients in our study was 42.33 years (s.d. 11.55), the proportion of women was ≈66% and the mean BMIs in the bariatric group and the matched group were 41.77 (s.d. 4.9) and 35.22 (s.d. 4.4), respectively. Comorbidities were present in both groups, with hypertension being the most common comorbidity (30% in the bariatric group and 33% in the matched group; *P* < 0.001), followed by diabetes and CKD. Statins and metformin were the most common medications prescribed in both groups. More diabetic patients were present in the bariatric group than in the matched group (18.3% *vs* 13.7%; *P* < 0.001) and patients in the bariatric group used more antidiabetic medications (Table 1). CRP and ESR levels were missing among the majority of subjects in both groups. The mean follow-up time from the index date until the end of the follow-up period was 7.31 years [s.d. 1.95; median follow-up 7.36 years (range 0–11)]. The number of patients with ≥5 years of follow-up was 18 143 (94.7%). Supplementary Fig. S1 shows the distribution of follow-up time among study population.

**Table 1:** Baseline characteristics of the study population.

Characteristics		Non-bariatric patients (*n* = 9583)	Bariatric patients (*n* = 9583)	Standard difference	*P*-value
Demographic					
Age at index date	Mean (s.d.)	42.33 (11.55)	42.33 (11.55)	<0.001	1
	Median (IQR)	42 (35–50)	42 (35–50)		
	Range	18–87	18–87		
Sex, *n* (%)	Male	3242 (33.8)	3242 (33.8)	<0.001	1
	Female	6341 (66.2)	6341 (66.2)		
SES, *n* (%)	Low	1261 (13.2)	959 (10)	0.109	<0.001
	Medium	5777 (60.3)	5788 (60.4)		
	High	2545 (26.6)	2836 (29.6)		
District, *n* (%)	North	2085 (21.8)	1936 (20.2)	0.212	<0.001
	Sharon	1321 (13.8)	2000 (20.9)		
	Centre	1906 (19.9)	1601 (16.7)		
	Jerusalem and Shfela	2286 (23.9)	2426 (25.3)		
	South	1985 (20.7)	1620 (16.9)		
Smoking status, *n* (%)	Ever	1355 (14.2)	1522 (15.9)	0.053	<0.001
	Never	5707 (59.9)	5521 (57.6)		
	Unknown	2472 (25.9)	2537 (26.5)		
BMI	Mean (s.d.)	35.22 (4.4)	41.77 (4.9)	2.134	<0.001
	Median (IQR)	34.7 (32–37.18)	40.89 (38.4–44.36)		
	Range	30–64	30–64		
	Missing	4593	54		
BMI (by median)	30–40	4411 (46)	3745 (39.1)	1.832	<0.001
	>40	579 (6)	5784 (60.4)		
	Missing	4593 (47.9)	54 (0.6)		
Chronic comorbidities, *n* (%)					
CHF	Yes	89 (0.9)	38 (0.4)	0.066	<0.001
CVD	Yes	127 (1.3)	69 (0.7)	0.06	<0.001
Diabetes	Yes	1311 (13.7)	1751 (18.3)	0.126	<0.001
Hypertension	Yes	2833 (30.1)	3160 (33)	0.062	<0.001
Cancer	Yes	417 (4.4)	288 (3)	0.072	<0.001
CKD	Yes	1245 (13)	1130 (11.8)	0.03	0.012
Diabetes type 1	Yes	5 (0.1)	21 (0.2)	0.045	0.002
Diabetes type 2	Yes	2199 (22.9)	2023 (21.1)	0.044	0.003
Cirrhosis	Yes	21 (0.2)	20 (0.2)	0.002	0.876
Dispensed medications					
Statins	Yes	2801 (29.2)	2585 (27)	0.05	<0.001
Metformin	Yes	1247 (13)	2191 (22.9)	0.259	<0.001
DPP-4i	Yes	177 (1.8)	326 (3.4)	0.097	<0.001
GLP-1a	Yes	63 (0.7)	252 (2.6)	0.156	<0.001
Labs					
Vitamin D	<25	2446 (25.5%)	5711 (59.6%)	0.748	<0.001
	>25	939 (9.8)	768 (8)		
	Missing	6198 (64.7%)	3104 (32.4%)		
HbA1c	<6.5	2237 (23.3)	5376 (56.1)	0.97	<0.001
	>6.5	852 (8.9)	1880 (19.6)		
	Missing	6494 (67.8)	2327 (24.3)		
HDL	<50 (female) <40 (male)	2237 (23.3)	5376 (56.1)	0.915	<0.001
	>50 (female) >40 (male)	852 (8.9)	1880 (19.6)		
	Missing	6494 (67.8)	2327 (24.3)		
LDL	<100	2310 (24.1)	2782 (29)	0.574	<0.001
	100–130	2288 (23.9)	3279 (34.2)		
	>130	1962 (20.5)	2618 (27.3)		
CRP	<0.3	939 (9.8)	486 (5.1)	0.289	<0.001
	>0.3	699 (7.3)	1418 (14.8)		
	Missing	7945 (82.9)	7679 (80.1)		
ESR	<20	1752 (18.3)	1712 (17.9)	0.22	<0.001
	>20	914 (9.5)	1616 (16.9)		
	Missing	6917 (72.2)	6255 (65.3)		

CHF: congestive heart failure; CVD: coronary vascular disease; DPP4i: dipeptidyl peptidase 4 inhibitor; GLP-1a: glucagon-like peptide-1 agonist.

Sleeve gastrectomy was the most prevalent bariatric surgery, performed in 7210 patients (74.3%), followed by adjustable gastric band surgery in 1505 patients (15.7%) and gastric bypass surgery in 942 patients (9.8%) (Supplementary Table S1).

### Incident RA

**Figure 2:**
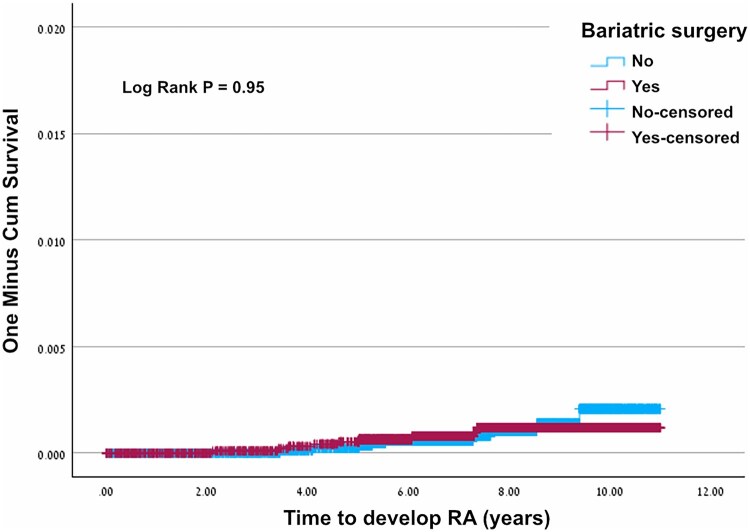
The comparative cumulative risk for incident RA between the bariatric surgery group and control group.

Among a total of 19 166 subjects (in both groups), 18 were diagnosed with incident RA during the follow-up period: 9 in the bariatric group (0.09%) and 9 in the control group (0.09%). The mean age of the patients with incident RA was 49.89 years and half of the patients were males (Table 2). There was no difference in baseline comorbidities or laboratory parameters between patients who developed RA and those who did not. We observed a trend toward higher CRP levels among patients who developed RA than among those who did not, but this difference was not statistically significant (*P* = 0.06). Bariatric surgery was not associated with a decreased incidence of RA during the follow-up period, as shown in Fig. 2 (logrank *P* = 0.95).

**Table 2:** Baseline characteristics of study patients stratified by RA diagnosis during follow-up.

Characteristics		No RA after index (*n* = 19 148)	RA after index (*n* = 18)	*P*-value
Demographic				
Bariatric, *n* (%)	Yes	9574 (50)	9 (50)	1
Age at index date	Mean (s.d.)	42.32 (11.55)	49.89 (12.21)	0.01
	Median (IQR)	42 (35–50)	49 (40.75–59.25)	
	Range	18–87	23–69	
Sex, *n* (%)	Male	6475 (33.8)	9 (50)	0.147
	Female	12 673 (66.2)	9 (50)	
SES, *n* (%)	Low	2218 (11.6)	2 (11.1)	0.94
	Medium	11 553 (60.3)	12 (66.7)	
	High	5377 (28.1)	4 (22.2)	
District, *n* (%)	North	4014 (21)	7 (38.9)	0.017
	Sharon	3319 (17.3)	2 (11.1)	
	Centre	3507 (18.3)	–	
	Jerusalem and Shfela	4704 (24.6)	8 (44.4)	
	South	3604 (18.8)	1 (5.6)	
Smoking status, *n* (%)	Ever	2872 (15)	5 (27.8)	0.23
	Never	11 218 (58.7)	10 (55.6)	
	Unknown	5006 (26.2)	3 (16.7)	
BMI	Mean (s.d.)	39.51 (5.66)	40.33 (5.81)	0.57
	Median (IQR)	39.16 (35.44–42.68)	39.74 (35.16–44.57)	
	Range	30–64	30–49.4	
	>30–≤40	8148 (42.6)	8 (44.4)	0.792
	>40	6356 (33.2)	7 (38.9)	
	Missing	4644 (24.3)	3 (16.7)	
Registries, *n* (%)				
CHF	Yes	127 (0.7)	–	1
CVD	Yes	196 (1)	–	1
Diabetes	Yes	3058 (16)	4 (22.2)	0.514
Blood pressure	Yes	6035 (31.5)	8 (44.4)	0.238
Cancer	Yes	705 (3.7)	–	0.65
CKD	Yes	2371 (12.4)	4 (22.2)	0.268
Diabetes type 1	Yes	26 (0.1)	–	1
Diabetes type 2	Yes	4220 (22)	5 (27.8)	0.57
Cirrhosis	Yes	41 (0.2)	–	1
Medications, *n* (%)				
Statins	Yes	5382 (28.1)	4 (22.2)	0.58
Metformin	Yes	3434 (17.9)	4 (22.2)	0.55
DPP-4	Yes	503 (2.6)	–	1
GLP-1	Yes	315 (1.6)	–	1
Labs				
Vitamin D	<25	8149 (42.6)	8 (44.4)	0.55
	>25	1707 (8.9)	–	
	Missing	9292 (48.5)	10 (55.6)	
HbA1c	<6.5	7604 (39.7)	9 (50)	0.61
	>6.5	2731 (14.3)	1 (5.6)	
	Missing	8813 (46)	8 (44.4)	
HDL	<50 (female) <40 (male)	8901 (46.5)	8 (44.4)	0.28
	>50 (female) >40 (male)	7403 (38.7)	5 (27.8)	
	Missing	2884 (14.9)	5 (27.8)	
LDL	<100	5090 (26.6)	2 (11.1)	0.1
	100–130	5564 (29.1)	3 (16.7)	
	>130	4572 (23.9)	8 (44.4)	
	Missing	3922 (20.5)	5 (27.8)	
CRP	<0.3	1425 (7.4)	–	0.06
	>0.3	2112 (11)	5 (27.8)	
	Missing	15 611 (81.5)	13 (72.2)	
PLT	<150	393 (2.1)	–	0.055
	150–450	16 512 (86.2)	13 (72.2)	
	>450	121 (0.6)	1 (5.6)	
	Missing	2122 (11.1)	4 (22.2)	
ESR	<20	3463 (18.1)	1 (5.6)	0.11
	>20	2525 (13.2)	5 (27.8)	
	Missing	13 160 (68.7)	12 (66.7)	

CHF: congestive heart failure; CVD: coronary vascular disease; DPP4i: dipeptidyl peptidase 4 inhibitor; GLP-1a: glucagon-like peptide-1 agonist.

### Multivariate analysis of the incidence of RA

To determine whether other factors were associated with the development of incident RA, we performed a multivariable Cox regression analysis including age, sex, smoking status, SES, blood pressure and diabetes status. Only age at the index date was associated with an increased risk for developing RA [HR 1.064 (95% CI 1.02, 1.110), *P* = 0.004] (Table 3). We also analysed the following BMI categories: median BMI >40 (group 1) and BMI >30–≤40 (group 2).

**Table 3:** Multivariable Cox regression analysis for bariatric surgery and HRs for prognostic factors for developing RA.

Variables	*P*-value	HR (95% CI)
Bariatric surgery (yes)	0.912	1.057 (0.396, 2.818)
Age at index	0.010	1.063 (1.015, 1.113)
Sex (female)	0.202	0.542 (0.211, 1.390)
SES (low)	0.697	
SES (medium)	0.960	1.039 (0.232, 4.661)
SES (high)	0.605	0.637 (0.115, 3.517)
Blood pressure	0.677	1.244 (0.446, 3.473)
Diabetes	0.671	1.307 (0.381, 4.480)

Multivariate Cox regression analysis (including age, sex, smoking status, SES, blood pressure, diabetes status and LDL levels) revealed an increased risk of incident RA in male patients with a BMI >40, as female sex was a protective factor in this group [HR 0.151 (95% CI 0.028, 0.826), *P* = 0.029]. No factors other than age affected the risk for incident RA, as shown in Table 4 and Supplementary Fig. S2.

**Table 4:** Multivariable Cox regression analysis for bariatric surgery and HRs for prognostic factors for developing RA in patients with BMI >40.

Variables	*P*-value	HR (95% CI)
Age at index	0.016	1.120 (1.022, 1.228)
Sex (female)	0.029	0.151 (0.028, 0.826)
SES (low)	0.918	
SES (medium)	0.779	0.730 (0.081, 6.602)
SES (high)	0.679	0.597 (0.051, 6.916)
Blood pressure	0.708	1.413 (0.232, 8.604)
LDL <100	0.355	
LDL 100–130	0.142	5.888 (0.553, 62.697)
LDL >130	0.072	9.525 (0.816, 111.152)
Diabetes	0.331	2.277 (0.434, 11.953)
Bariatric surgery (yes)	0.322	0.330 (0.037, 2.970)

### The effect of changes in BMI on RA incidence

Changes in BMI during the follow-up period were calculated as [(BMI at the end of follow-up − baseline BMI)/baseline BMI] * 100. The majority of patients in the bariatric group did not gain weight after the follow-up period (79.7%) and only 12.3% gained weight at the end of the follow-up period (Supplementary Table S2).

To determine whether changes in BMI during follow-up rather than bariatric surgery were associated with the development of RA, we performed another multivariable Cox regression analysis including BMI change during the follow-up period, age, sex, smoking status, SES, blood pressure and diabetes status. Changes in BMI during the follow-up time were not associated with the development of RA in the cohort [HR 0.63 (95% CI 0.18, 2.15), *P* = 0.45].

## Discussion

In this large, population-based retrospective cohort study, we evaluated whether bariatric surgery and its associated substantial weight loss influence the subsequent risk of developing RA. Despite epidemiological evidence linking obesity to increased RA incidence, particularly among women and seronegative phenotypes—our findings demonstrate that bariatric surgery does not confer a measurable reduction in incident RA over an average of 7.3 years of follow-up. The incidence of RA was identical in the surgical and matched non-surgical cohorts (0.09%) and bariatric surgery did not emerge as a protective factor in multivariable modelling.

Overall, except for age at bariatric surgery, the incidence of RA among obese patients was not associated with other factors, including BMI changes during follow-up, SES, smoking status or baseline comorbidities. However, among male patients with a BMI >40, an increased risk for incident RA was observed.

The association between age at bariatric surgery and RA incidence was anticipated since the mean age of the patients in our study cohort was 42.33 years (s.d. 11.55) and RA occurs in middle-aged and older individuals with peak incidence rates between the ages of 65 and 80 years [33].

To date, only one smaller-scale study, the Swedish Obese Subjects (SOS) study, has assessed the effect of bariatric surgery on the incidence of RA and found that weight loss secondary to bariatric surgery was not associated with a reduced risk of developing RA [32]. These results are in line with our study results; however, the SOS study had several notable limitations: the primary endpoint in the Swedish register was mortality rather than incident RA, the study population originated exclusively from surgical departments, limiting generalizability, and a significant proportion (14%) of the control group eventually underwent bariatric surgery.

Our study overcomes several of these limitations by leveraging the comprehensive MHS database, allowing a much larger patient cohort (nearly 20 000 subjects) and the use of stringent RA case definitions, a matched control population with stable BMI ≥30 that did not cross over to surgery. Thus our findings substantially reinforce and extend the evidence that weight loss induced by bariatric surgery does not modify the risk of RA incidence.

The lack of a protective effect from bariatric surgery should not be interpreted as evidence against obesity being a relevant factor in RA pathogenesis. Rather, it suggests that weight loss alone is insufficient to counteract the underlying mechanisms driving RA initiation, which likely involve a complex interplay of genetic susceptibility, environmental triggers, epigenetic regulation and immunologic events.

One way to explain these results is that bariatric surgery and weight loss do not alter other risk factors for developing RA, such as age, genetic predisposition, smoking status and family history. These risk factors might have a more significant effect on the risk of developing RA than obesity alone.

In addition, increased adipose mass is associated with increased expression of pro-inflammatory cytokines (such as TNF-α, IL-1, IL-6 and IL-8) and altered production of adipokines [34–37]. Circulating levels of leptin and pro-inflammatory cytokines are significantly decreased after bariatric surgery [38]. Baseline levels of adipokines, especially leptin, have been shown to predict radiographic progression in early RA [37], but it is not known whether adipokines contribute to the initiation of RA. Thus changes in their levels after bariatric surgery might affect the RA disease course but not modify the risk for RA development.

Moreover, the reduction in pro-inflammatory markers following bariatric surgery might contribute to the association between bariatric surgery and lower RA activity, decreased serum inflammatory markers and less RA-related medication use in patients with a precedent RA diagnosis [12, 30, 31]. However, we suppose that these effects are not significant for preventing the development of RA disease since obesity is not the main or the only risk factor for RA development.

Our study has several limitations. First, the study was retrospective in design. Second, the mean age of our study population was younger than the typical age of RA onset, a fact that might limit our ability to detect any possible effect of bariatric surgery on the risk for developing RA. Third, the follow-up period was relatively short; the mean follow-up of 7.31 years is robust for many metabolic endpoints, but RA pathogenesis often involves a decade or more of preclinical autoimmune activity before the onset of symptoms. Fourth, we relied on data collected during routine clinical practice and not all data, such as BMI, were available for the years following bariatric surgery. To address some of these limitations, we aim to report the results of a longer follow-up period. We believe that more cases of RA will be detected as the population of the study ages, increasing the odds of detecting any possible true protective effect of bariatric surgery on the risk of developing RA.

In conclusion, we observed no association between bariatric surgery and incident RA among obese patients during a mean follow-up of 7.3 years. Further longitudinal studies with longer follow-up periods are needed to confirm or reject these findings.

## Supplementary Material

rkag025_Supplementary_Data

## Data Availability

The data that support the findings of this study are available from the MHS, but restrictions apply to the availability of these data, which were used under licence for the current study and so are not publicly available. However, the data are available from the authors upon reasonable request and with the permission of the MHS.
