# Supplementary material for: The association between bariatric surgery and incident rheumatoid arthritis
Source: Rheumatol Adv Pract. 2026 Feb 18;10(2):rkag025. doi: 10.1093/rap/rkag025 (PMC13032893; doi:10.1093/rap/rkag025)
Supplement: rkag025_Supplementary_Data [file rkag025_supplementary_data.zip › 25-178 Supplementary Figures.docx]

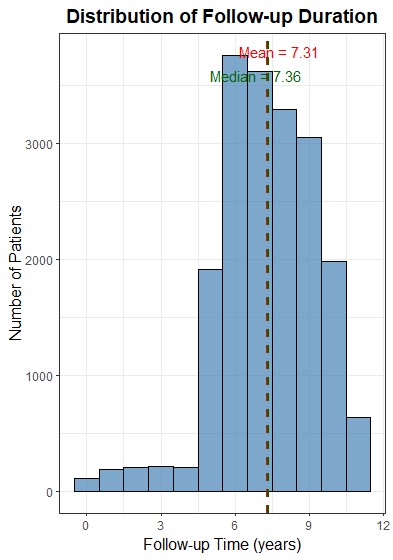


**Supplementary Figure S1.** Distribution of follow-up duration.

ALT TEXT: Histogram showing distribution of follow-up duration in years among study participants, with most observations between five and nine years; vertical lines indicate mean follow-up of 7.31 years and median of 7.36 years.

**

**

**Supplementary Figure S2.** The comparative cumulative risk for incident RA between male and female patients among patients with 30<BMI<40 (A), and BMI≥40 (B). RA, rheumatoid arthritis.

ALT TEXT: Two-panel Kaplan–Meier plots comparing cumulative incidence of rheumatoid arthritis between male and female patients, stratified by body mass index 30 to less than 40 in panel A and 40 or higher in panel B, showing higher cumulative risk among males in the highest body mass index group.
